# Animal Dietary Exposure to Methylxanthines through the Inclusion of Former Food Products (FFPs) in Feed

**DOI:** 10.1021/acsagscitech.5c01031

**Published:** 2026-02-05

**Authors:** Francesca Mercogliano, Chiara Di Lorenzo, Marco Tretola, Corinne Bani, Michele Manoni, Luciano Pinotti

**Affiliations:** 1 Department of Pharmacological and Biomolecular Sciences, 9304 Università degli Studi di Milano, via Balzaretti 9, Milano 20133, Italy; 2 Coordinated Research Center (CRC) “Innovation for Well-Being and Environment”, 9304 Università degli Studi di Milano, Milan 20133, Italy; 3 Institute for Livestock Sciences, Agroscope, La Tioleyre 4, Posieux 1725, Switzerland; 4 Department of Veterinary Medicine and Animal Sciences, 9304 Università degli Studi di Milano, via dell’Università 6, Lodi 26900, Italy

**Keywords:** ex-food, theobromine, alkaloids, food sustainability, food losses and food waste

## Abstract

Former food products (FFPs) are increasingly recognized as sustainable feed ingredients. While nutritionally valuable, FFPs may contain cocoa-based confectionery, which is a source of methylxanthines such as theobromine (TB) and caffeine (CF) and can impact animal health. This study quantified TB and CF concentrations in 12 FFPs using HPLC-UV, evaluated FFP inclusion rates in animals’ diets against European Union (EU) maximum levels (MLs), and dietary exposure against toxicological thresholds. TB levels ranged from 59.6 to 1147.1 μg/g and CF from 9.3 to 118.1 μg/g. All products, except one, complied with EU MLs when included at 30% in the diet (on a dry basis). Modeled animal dietary exposure (ADE) indicated that, in most proposed species, TB intake was below safety thresholds; however, the maximum exposure scenarios in piglets exceeded reported no-observed adverse effect levels (NOAEL). These findings highlight the need for species-specific and production-stage-specific evaluations and accurate quantification of methylxanthines when formulating diets with FFPs.

## Graphical abstract

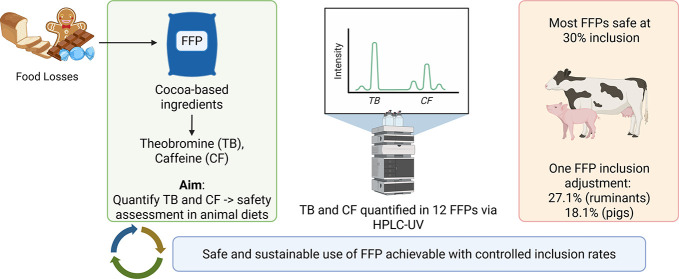

## Introduction

In recent years, the global agri-food system has faced growing pressure to adopt more sustainable and circular production models in response to food waste, environmental degradation, and resource scarcity.^1^ In September 2015, as part of the 2030 Sustainable Development Goals, the United Nations General Assembly adopted a target of halving per capita food waste at the retail and consumer level and reducing food losses along production and supply chains.^2^ One of the central promoted strategies is the valorization of food losses and food waste (FLW) through their reintegration into productive chains. In this context, former food products (FFPs) refer to foodstuffs originally intended for human consumption but, due to various nonsafety-related reasons such as packaging errors, aesthetic flaws, or logistical oversupply, are no longer marketable for humans (consolidated text of Commission Regulation (EU) No. 68/2013).^3^ Despite their removal from the retail food chain, FFPs often retain a high nutritional value, including significant levels of starch, sugars, and fats, making them promising alternative feed ingredients.^4^ FFPs may include different processed food items such as bread, pasta, biscuits, snacks, and confectionery products.^5^

These materials, although technically safe for consumption, are often discarded by the food industry due to visual or packaging defects, as the costs associated with repackaging or reprocessing exceed their commercial value. While these items may still be safe for consumption, they often fail to reach consumers due to logistical challenges or regulatory constraints.^6^ In 2018, the European waste legislation was amended and announced new measures for waste prevention, including the exclusion of materials of nonanimal origin destined as feed from its scope.^7,8^ The amendment reduced the regulatory burden on operators by removing the need to comply with waste legislation for food of nonanimal origin intended for feed.^9^ This facilitates the reuse of such materials, supports circular economy practices, and eliminates legal inconsistencies among European Member States. Given this context, the integration of FFPs into animal feed has emerged as a promising circular economy strategy.

According to Commission Regulation (EU) No. 68/2013 on the Catalogue of Feed Materials, former foods (e.g., products from the bakery, pasta, pastry, and confectionery industry and breakfast cereal manufacture) are explicitly listed in the Catalogue of Feed Materials. However, FFPs may contain compounds naturally present in the original food matrix, which can represent an area of concern, particularly when FFPs include chocolate- and cocoa-based confectionery, which are well-documented sources of methylxanthines such as theobromine (TB) and caffeine (CF). These compounds can exert bioactive effects on animals, potentially affecting the cardiovascular, nervous, and gastrointestinal systems.^10–16^ The primary mechanism of action for methylxanthines has long been recognized as the inhibition of adenosine receptors. Interestingly, theobromine exhibits significantly lower affinity for adenosine receptors compared to caffeine.^17^ Nevertheless, caffeine and theobromine display varying affinities for different adenosine receptor subtypes, which may help explain why these two compounds produce distinct physiological effects.^12^ In particular, TB has stronger coronary vasodilator and cardiac stimulation effects than CF, while CF is a stronger central nervous system, respiratory, and skeletal muscle stimulant and has mildly stronger diuresis effects than TB.^10^

In the European Union (EU), animal feed is considered noncompliant if the concentration of undesirable substances exceeds the maximum levels (MLs) specified in Annex I of Directive 2002/32/EC of the European Parliament and Council, as last amended in 2019 (Art. 3.2).^18^ TB is classified among these undesirable substances (Annex I, Directive 2002/32/EC). When this Directive first entered into force in May 2022, it reported generally higher theobromine EU MLs than the MLs in force nowadays. The MLs were 300 mg/kg in complete feedstuffs, with the exception of feed intended for adult cattle, which had an even higher ML of 700 mg/kg. After the EFSA CONTAM Panel highlighted that existing TB MLs may be insufficient to safeguard certain species,^19^ the Commission decided to lower TB MLs.^20^ To date, the current ML is 300 mg/kg for complete feed with 12% moisture content, except for pigs, where the limit is 200 mg/kg, and for dogs, rabbits, horses, and fur animals, where the limit is set at 50 mg/kg. Unlike TB, Directive 2002/32/EC does not include caffeine in the list of undesirable substances in feed, although the ingestion of caffeine-rich coffee coproducts (e.g., coffee husks and coffee pulp) has shown undesirable effects, including a decrease in weight gain, feed conversion, and metabolized energy in poultry^21^ and toxicosis cases given by coffee husks in horses.^22^

From a nutritional perspective, FFPs are highly variable, although former foodstuff processors have established methods to produce consistent commercial products.^23^ FFPs are characterized by high energy density, abundant simple carbohydrates, and elevated fat content due to their processed nature. These properties make them particularly suitable for inclusion in livestock diets under controlled conditions.^4,24^ Studies have shown no adverse effects on gastrointestinal health, microbiota, growth performance,^25^ and meat quality,^26^ and no undesirable effects on physiology and metabolism, especially considering the plasma metabolome^27^ and liver proteome^28^ from postweaning to finishing pigs fed diets partially replacing common feed materials with FFP. Additionally, FFPs, even in combination with cocoa shells, did not impair the metabolic health or ruminal fermentation of early lactating cows^29^ and did not cause any significant difference in terms of feed intake, body condition score, milk yield, and milk quality in lactating buffaloes.^30^ Overall, from a nutritional point of view, FFPs may help reduce food and feed competition without compromising animal productivity and health. However, their use requires careful regulation of composition and inclusion rate into the animal diet.^31^ In addition to nutritional adequacy, the sustainability implications of using FFPs as feeds are significant. Life cycle assessments indicate that diverting FFPs to animal feed conserves water and reduces environmental burdens^32^ compared to alternative disposal methods such as the production of electricity, heat, and digestate.^33^ From a feed safety perspective, FFPs must comply with comprehensive feed regulatory frameworks. Specifically, Regulation (EC) No. 183/2005 mandates that all feed business operators, including former foodstuff processors, implement Hazard Analysis and Critical Control Point (HACCP) principles to ensure feed hygiene and safety throughout the production chain.^34^ Additionally, FFPs must adhere to Regulation (EC) No. 178/2002, which establishes general principles of food law, including traceability and the obligation to withdraw unsafe products from the market. Moreover, Regulation (EC) No. 767/2009 ^35^ on the placing on the market and use of feed also fully applies to former foodstuff processors, outlining essential requirements, including accurate and transparent labeling, claims, and conditions of use, ensuring that end users receive safe, appropriately described products.^36^ Regulatory frameworks, such as those established following the Bovine Spongiform Encephalopathy (BSE) crisis, restrict the use of certain waste-derived materials, especially those containing animal byproducts.^37^ Additionally, the boundary between feed-eligible surplus and outright waste is tightly regulated, with postconsumer waste (e.g., leftovers from canteens or households) excluded from animal feed chains.^7,38^

In this study, the aim was to investigate the presence of TB and CF in 12 FFPs, from FFP processors based in different EU countries, to derive dietary exposure to target animals and draw safety considerations on FFP implementation in animals’ diets based on methylxanthine content. This assessment is particularly relevant in the context of Directive 2002/32/EC, which establishes species-specific thresholds and the maximum levels for undesirable substances in animal feed, including TB, to guarantee animal health and feed safety.

## Materials and Methods

### Chemicals

Caffeine and theobromine standards were purchased from Sigma-Aldrich (Merck, Steinheim, Germany). Methanol, ethanol, HPLC-grade water, acetonitrile, acetone, toluene, *n*-hexane, ethyl acetate, reagents for analysis, and acids were purchased from VWR International (Fontenay-sous-Bois, France).

### Samples

Twelve FFP samples (Figure 1) were collected within the framework of a voluntary campaign conducted across European former food processors to collect FFPs and contribute to the development of a realistic overview of FFPs currently available on the market for feed applications. FFP1, FFP2, and FFP3 consist of sweets, baked products, pastry items, and cereal products such as wheat flour and bran. FFP4 includes puffed and extruded rice and corn cakes, while FFP5 comprises different products from the food industry, including pasta, baked goods, sweets, and pastry products. FFP6 and FFP10 primarily contain bakery products, including biscuits. FFP7, FFP8, and FFP9 derive from confectionery items and food byproducts. FFP11 includes bread, pasta, and savory baked snacks, while FFP12 includes chocolate and biscuits, and it is formulated for piglets. In addition to the FFPs, a negative control (NC) containing no former food material and composed of a conventional postweaning piglet diet (see Mazzoleni et al.^25^), and a positive control (PC) composed of cocoa shells, as expected to contain TB and CF, were included in this study. All samples were milled using a 1 mm screen mill (Model 160-D, Jacobsen Machine Works, Minneapolis, MN, USA) and stored at 4 °C until analysis.

**Figure 1:**
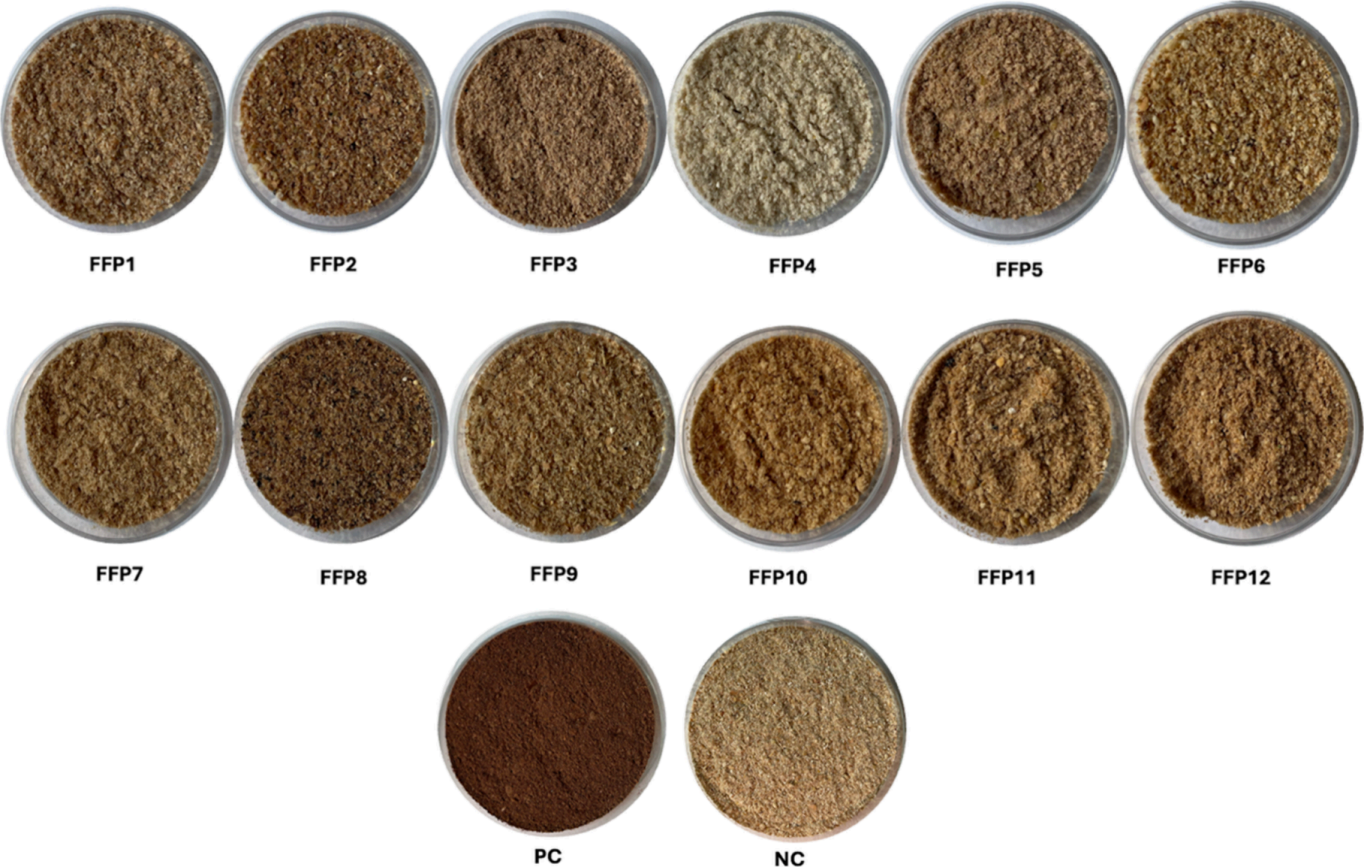
FFP samples (FFP1-FFP12), positive control (PC) and negative control (NC).

### Chemical Composition Analysis

Samples were analyzed for moisture, starch, sugars, crude protein (CP), ether extract (EE), neutral detergent fiber (NDF), and ash using standardized procedures based on the Association of Official Analytical Chemists^39^ and European Commission regulation 152/2009.^40^ Samples FFP1-FFP6 were previously analyzed in the context of other studies.^41,42^ Moisture content was determined by oven-drying at 130 °C for 2 h, following Commission Regulation No. 152/2009. Results were expressed on a dry matter (DM) basis. Starch was measured using a polarimetric method as per European Commission guidelines,^40^ and sugar content was determined according to the official method outlined in the same regulation. Crude protein was analyzed using the Kjeldahl method 2001.11.^39^ Ether extract was determined via the Soxhlet method after acid hydrolysis, following Commission Regulation No. 152/2009.^40^ Neutral detergent fiber (NDF) was analyzed using an Ankom 220 fiber analyzer (ANKOM Technology, Fairport, NY, USA) according to AOAC method 2002.04, with the use of heat-stable amylase, and results were expressed without including residual ash (aNDFom). Ash content was measured by combustion in a muffle furnace at 550 °C^39^ (method 942.05).

### Extraction Method for the Chromatographic Techniques

One gram of each ground sample was defatted by mechanical stirring with 5 mL of *n*-hexane, repeated four times until clear. After defatting, 10 mL of an 80:20 (v/v) methanol:water solution was added to the samples for overnight extraction under continuous stirring by a rotary plate (ASAL srl, Milano, Italia). The samples were centrifuged at 3000*g* at 4 °C for 15 min (Centrifuge 5810R, Eppendorf, Hamburg, Germany). The resulting extract was filtered through a 0.45 μm PTFE filter (VWR, Fontenay-sous-Bois, France) and stored at −20 °C until analysis.

### High-Performance Thin-Layer Chromatography Analysis

High-performance thin-layer chromatography (HPTLC) is a chromatographic technique that allows a relatively fast, accurate, and cost-efficient detection of compounds.^43^

In this study, HPTLC was used for the initial screening of CF and TB in the FFP samples. Extracted samples were dried by nitrogen flow and suspended in methanol. Ten μL of samples (2.0 g/mL) and standards of CF and TB (0.5 mg/mL) were loaded onto HPTLC silica-gel plates 60 F254 (dimensions: 10 × 20 cm, manufacturer: Merck, Darmstadt, Germany) with a semiautomatic sample applicator (Linomat 4, CAMAG, Muttenz, Switzerland). The mobile phase consisted of 10 mL of water:methanol:ethyl acetate 0.7:1.1:8.2, *v*/*v*/*v*. The chromatographic run was revealed at 254 nm (Software VisionCats, CAMAG, Muttenz, Switzerland).^43^

### High-Performance Liquid Chromatography Analysis

High-performance liquid chromatography coupled with ultraviolet detection (HPLC-UV) is a widely utilized analytical technique for quantifying compounds that absorb UV light. In this study, the quantification of TB and CF was conducted following the procedure described by Mercogliano and colleagues,^44^ with minor adaptations for the present matrices. Chromatographic separations were performed using a reversed-phase YMC-Triart C18 column (250 mm length, 3.0 μm particle size). The HPLC system (Jasco, Tokyo, Japan) included two PU-1580 pumps, a DG-2080-54 degasser, an AS-2059 Plus autosampler, a UV-975 UV detector, and an LC-NETII/ADC interface coupled with a Rheodyne injection valve (Cotati, CA, USA) equipped with a 100 μL loop. Data acquisition and processing were carried out by using ChromNAV software (Jasco, Tokyo, Japan). The mobile phases consisted of (A) 0.5% (v/v) formic acid in water and (B) 0.5% (v/v) formic acid in acetonitrile. Gradient elution was applied at a flow rate of 1.0 mL/min as follows: 0–30 min: 90–75%, A; 30–35 min: 75–0% A; 35–39 min: 0% A isocratic; 39–40 min: 0–90% A; and 40–50 min: 90% isocratic A. The UV detector was set at 280 nm. Standard stock solutions were prepared at a concentration of 500 μg/mL in methanol:water (80:20, v/v). Working standard solutions were obtained by spiking the blank sample (showing no interferences at analyte retention time) to achieve final concentrations of 0.5–50 μg/mL for TB and 0.2–4 μg/mL for CF. Acceptance criteria for linearity were *R* ^2^ ≥ 0.99. Solutions were stored at −20 °C until use. The limit of detection (LOD) and quantification (LOQ) were confirmed at a signal-to-noise ratio of 3 and 10, respectively.^45^ Samples were prepared as described in the extraction section and analyzed in triplicate as such or diluted with methanol:water (80:20, v/v) prior to injection.

### Animal Dietary Exposure Assessment

Animal dietary exposure (ADE) to TB and CF was calculated based on default values for animal weight and feed intake used by the EFSA (European Food Safety Authority) Panels on Additives and Products or Substances used in Animal Feed (FEEDAP) and by the Panel on Contaminants (CONTAM).^46,47^

After the quantification of TB and CF in the samples, the corresponding concentration per kilogram of DM feed was calculated as follows:

$$C_{\mathrm{feed}},\mathrm{DM}=\frac{C_{\mathrm{sample}}}{\left(1-\frac{{\mathrm{moisture}}_{\mathrm{FFP}}\;}{100}\right)}\times \frac{\mathrm{IR}}{100}$$ 1

where *C*_feed_, DM (mg/kg DM) is the concentration of TB or CF per kilogram of complete feed in a DM basis, *C*_sample_ (μg/g) is the concentration of TB or CF measured in the FFP, moisture_FFP_ (%) is the moisture in the FFP, and IR (%) is the inclusion rate of the FFP in feed on a DM basis.

According to the available literature, FFPs have been proposed as partial substitutes for conventional feed ingredients, with inclusion levels up to 30% (on DM basis) in the diets of ruminants and pigs, not representing a risk mainly in terms of growth performance,^25,29,48,49^ product quality,^31^ gut health and physiology,^26,50,51^ and environmental impact.^31,32,50,51^ This level of substitution was therefore considered for the ADE calculations in this study. Ruminants^24,29–31,48,49^ and pigs^25,52^ were selected as target animal categories since the use of FFPs has been specifically proposed for these animal species, while the literature data on their inclusion in poultry diets remain very limited.^53^ In addition, due to the established susceptibility of dogs and horses to theobromine toxicity, cocoa byproducts and confectionery residues are generally not incorporated into their feeds,^19^ and therefore, these species were not considered as target animals in this study.

Four scenarios were then evaluated by using the lowest and highest measured TB or CF occurrence values and the median and the mean values across all FFP samples.

The ADE was calculated as follows:

$$\mathrm{ADE}=\left(\frac{C_{\mathrm{feed}},\mathrm{DM}\times \mathrm{FI}}{\mathrm{bw}}\right)$$ 2

where ADE (mg/kg of bw/day) is the animal dietary exposure, *C*_feed_, DM (mg/kg DM) is the concentration of TB or CF per kilogram of complete feed on DM basis, FI (kg/day DM) is the feed intake on DM basis, and bw is the body weight.

The modeled ADE values, derived from the inclusion of FFPs in animals’ diets, were compared with published and derived NOAEL and LOAEL (no-observed adverse effect; lowest-observed adverse effect) values to evaluate potential toxicological risks.

### Statistical Analysis

Descriptive statistical analyses of HPLC results (mean values, standard deviation), Pearson correlation coefficient (*R* ^2^), and Kruskal–Wallis were performed using IBM SPSS Statistics for Macintosh, software 29.0.2.0 (IBM Corp, Armonk, NY, USA). Graphs were generated using GraphPad Prism for MacOS, version 10.3.1. Normality was assessed using the Shapiro–Wilk test, and homogeneity of variances was evaluated with Levene’s test. Given the nonnormality, the nonparametric Kruskal–Wallis was used and significant differences were determined by *post hoc* Bonferroni, with the significance set at *p* < 0.05.

## Results and Discussion

### Chemical Composition

The nutritional composition of the FFPs (Table 1) demonstrated variability across the samples. Moisture content ranged from 6.2 to 8.7%. Starch concentrations varied markedly, with FFP4 containing mainly rice and corn cakes, reaching 73.4%, while FFP12, which contained biscuits, had the lowest value at 38.6%. Sugar content was generally high in FFPs, reaching a maximum of 27.3% in FFP6, consistent with the literature, which highlights high simple sugar levels in ex-foods.^50^ Crude protein averaged approximately 10%, comparable to cereal grains, such as wheat, although FFP12 exhibited higher protein levels (up to 19.3%), in line with the PC (19.0%), both intentionally produced for piglets. Ether extract values ranged from 4.8 to 15.4%, confirming that FFPs are lipid-enriched compared to standard cereal-based feeds.^41^ Fiber content was heterogeneous, consisting of crude fiber ranging between 0.6 and 5.5%, while NDF ranged from 5.4 to 32.1%, with FFP8 and FFP12 presenting the highest NDF concentrations. Crude ash content varied from 1.4 to 5.5%. These compositional profiles, particularly elevated starch, fat, and digestible carbohydrate, support the classification of FFPs as a “fortified version of common cereal grains”.^52^ Such characteristics make FFPs promising energy-dense feed ingredients, although variability across samples suggests careful nutritional considerations.

**Table 1:** Nutrient Composition of FFPs (% DM Basis)

sample	moisture	starch	sugars	crude protein	ether extract	crude fiber	neutral detergent fiber	ash
FFP1	7.4	45.9	25.7	9.2	11.7	2.7	10.1	2.2
FFP2	8.7	42.3	22.1	11.5	11.0	4.4	15.7	3.2
FFP3	8.5	44.6	20.1	12.0	9.9	4.5	16.2	3.4
FFP4	8.1	73.4	12.4	9.1	4.8	1.9	5.4	1.4
FFP5	8.7	56.4	20.2	10.9	10.4	1.5	7.7	2.1
FFP6	6.2	51.5	27.3	7.3	12.9	1.3	5.6	1.8
FFP7	7.8	42.6	11.2	11.7	11.7	3.3	21.9	3.5
FFP8	8.7	40.6	12.3	12.9	8.0	5.5	32.1	3.7
FFP9	8.5	40.3	10.4	12.8	10.1	4.1	25.5	3.8
FFP10	6.9	49.0	20.0	9.4	15.4	0.6	8.5	1.9
FFP11	7.3	51.5	14.9	9.5	12.5	0.7	16.3	3.5
FFP12	8.7	38.6	11.7	19.3	6.1	3.1	28.7	5.5
PC	10.4	<3.2	1.0	19.0	8.9	16.2	48.6	8.2
NC	9.9	39.9	4.69	19.1	3.9	3.1	11.2	6.1

### Screening for Theobromine and Caffeine by HPTLC

HPTLC was employed for quick qualitative screening to identify the possible presence of TB and CF in FFPs. The chromatographic analysis (Figure 2) revealed distinct retention factor (Rf) values for both TB (Rf = 0.40) and CF (Rf = 0.46), enabling their identification based on comparison with known standards. In this preliminary screening, TB was identified in 11 FFP samples among the 12 analyzed and was not detected in FFP4 and the NC. CF was only clearly visible in the PC. Given these results, quantification of TB and CF was performed by HPLC for more sensitive and quantitative analysis.

**Figure 2:**
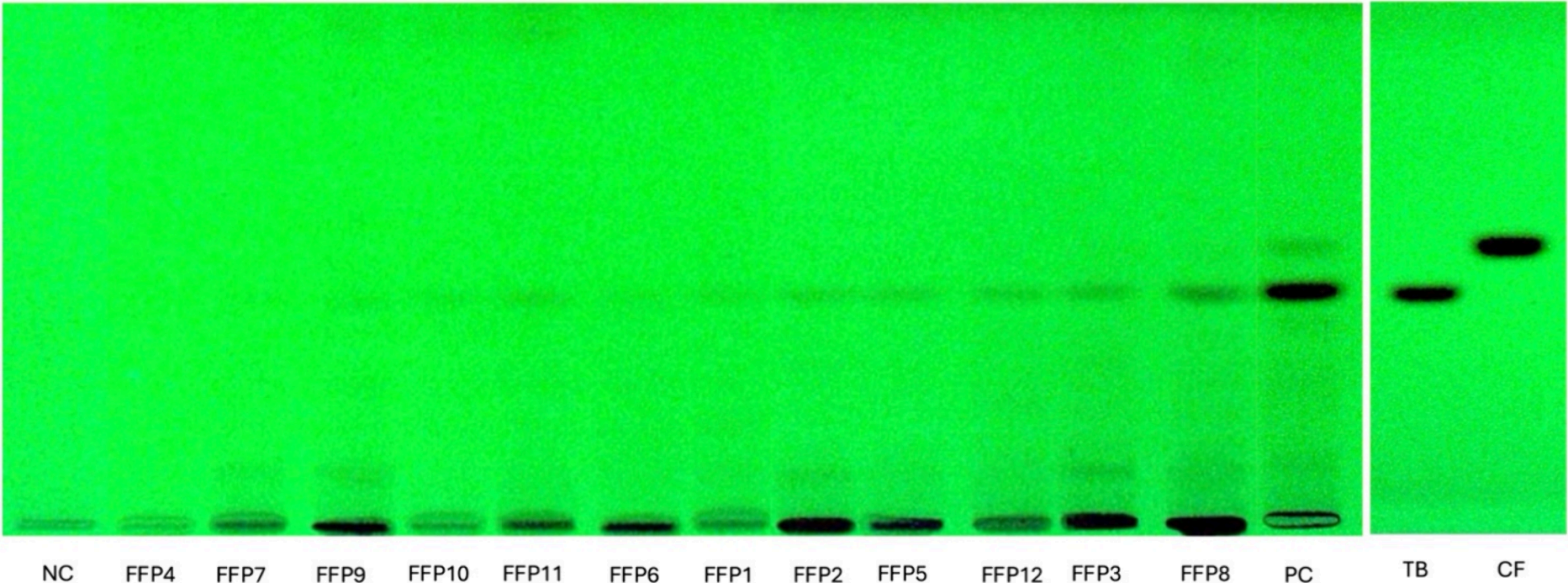
HPTLC plates detected at 254 nm. TB: theobromine; CF: caffeine.

### Quantification of Theobromine and Caffeine in FFPs and Resulting Concentrations in Feed

HPLC-UV analysis was carried out to quantify TB and CF across the FFP samples. The chromatographic profiles, shown in Figure 3, displayed a first integrated peak, corresponding to TB, at a retention time (Rt) of 6.8 min and a second integrated peak corresponding to CF with an Rt of 13.6 min in all FFP samples and PC, while no peaks were detectable in the NC.

**Figure 3:**
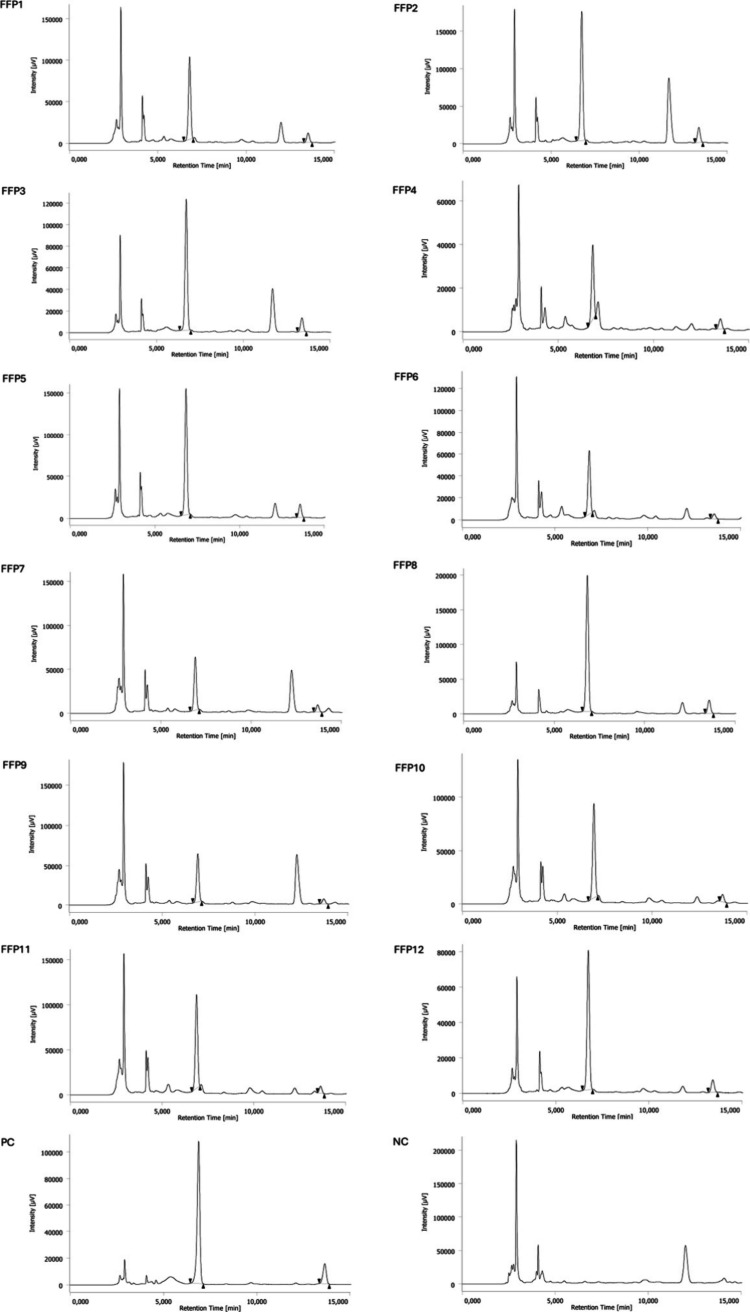
Chromatograms of FFP samples, PC and NC. The first integrated peak corresponds to TB, and the second integrated peak corresponds to CF.

Quantitative results of TB and CF, reported in Table 2, are expressed as the mean ± standard deviation (μg/g) and TB/CF ratio.

**Table 2:** Content of TB and CF in FFPs^a^

sample	TB (μg/g)	CF (μg/g)	TB/CF ratio	IR%	TB in feed (mg/kg of feed 12% DM)	CF in feed (mg/kg of feed 12% DM)
FFP1	187.45 ± 2.29^abc^	23.34 ± 0.74^abc^	8.0	30%	53.44	7.00
FFP2	329.57 ± 4.02^abc^	40.91 ± 1.85^abc^	8.1	30%	95.30	12.27
FFP3	458.11 ± 7.80^bc^	52.31 ± 2.22^bc^	8.8	30%	132.18	15.69
FFP4	59.59 ± 1.46^a^	9.97 ± 0.49^a^	6.0	30%	17.12	2.99
FFP5	293.52 ± 5.99^abc^	32.33 ± 1.14^abc^	9.1	30%	84.87	9.70
FFP6	102.92 ± 4.37^ab^	9.28 ± 0.43^a^	11.1	30%	28.97	2.78
FFP7	113.87 ± 4.87^abc^	17.25 ± 0.52^abc^	6.6	30%	32.60	5.17
FFP8	1147.08 ± 21.95^c^	118.08 ± 2.39^c^	9.7	27.1%*	300.00	30.88
				18.1%**	200.00	20.59
FFP9	124.94 ± 6.66^abc^	13.32 ± 0.25^abc^	9.4	30%	36.05	4.00
FFP10	162.87 ± 6.02^abc^	13.11 ± 0.43^abc^	12.4	30%	46.18	3.93
FFP11	190.61 ± 10.19^abc^	15.14 ± 0.65^abc^	12.6	30%	54.28	4.54
FFP12	298.71 ± 6.78^abc^	30.77 ± 0.77^abc^	9.7	30%	86.38	9.23
PC	7115.48 ± 261.19	1105.66 ± 16.41	6.4			
NC	<LOD	<LOD				

a Expressed as mean ± SD (μg/g), *n* = 3, values with the different letters are statistically significant (*p* < 0.05) within the FFP samples. LOD for TB: 0.03 μg/g, LOD for CF: 0.1 μg/g. IR%: selected inclusion rate, TB/CF: TB and CF ratio, TB and CF in feed: resulting mg of TB and CF concentrations per kilogram of feed with a moisture content of 12%. *Resulting inclusion rate for not exceeding the EU ML (Directive 2002/32/EC) of TB in complete feed for ruminants (300 mg/kg). **Resulting inclusion rate for not exceeding the EU ML (Directive 2002/32/EC) of TB in complete feed for pigs (200 mg/kg).

The detection of TB and CF in all tested FFPs suggests the presence of ingredients containing methylxanthines within these products. Among purine alkaloids, theobromine is the principal compound in cocoa and chocolate products.^54^ The TB/CF ratios observed (6.0–12.6) are consistent with cocoa and/or chocolate-derived products^12,55^ and clearly differ from those of other methylxantine-containing products like coffee, teas, and guarana, in which CF predominates over TB.^12,56^ While minor contributions from these sources cannot be excluded, the alkaloid profile is most consistent with cocoa- and chocolate-derived products and is therefore the likely principal source of these compounds in the samples.

Among FFPs, TB concentrations ranged from 59.59 ± 1.46 μg/g in FFP4 to 1147.08 ± 21.95 μg/g in FFP8, exhibiting substantial variability. CF concentrations ranged from 9.28 ± 0.43 μg/g in FFP6 to 118.08 ± 2.39 μg/g in FFP8. This variability may reflect both differences in raw material composition and the relative presence of chocolate or cocoa ingredients within each product category. FFP4 and FFP6 showed the lowest TB and CF contents, respectively. FFP4, composed of puffed and extruded rice and corn cakes, was not expected to contain cocoa-derived compounds, and the trace amounts observed may reflect cross-contamination during processing. Similarly, FFP6, derived from bakery and confectionery items, showed low levels of TB and CF, suggesting cross-contamination or limited inclusion of cocoa-based ingredients. In contrast, FFP8, deriving from confectionery products and food byproducts, exhibited the highest concentrations of both alkaloids, indicating a potential high cocoa content in this product. Intermediate concentrations were observed in mixed-composition samples, including FFP1, FFP2, and FFP3, which included a combination of sweets, baked products, and cereal items, with TB ranging from 187.45 to 458.11 μg/g and CF from 23.34 to 52.31 μg/g. Declared chocolate-containing FFP12 showed moderately elevated levels (298.71 ± 6.78 μg/g TB, 30.77 ± 0.77 μg/g CF), comparable to those measured in FFP5, a product containing pasta, baked goods, sweets, and pastry products. FFP6 and FFP10, containing bakery products and biscuits clustered in lower ranges (102.92–162.87 μg/g for TB and 9.28–13.11 μg/g for CF). Other confectionery-derived samples (FFP7 and FFP9) displayed relatively low concentrations (113.87–124.94 μg/g TB, 13.32–17.25 μg/g CF) compared to FFP8, suggesting variability and heterogeneity of cocoa content across different confectionery products. This variability may also reflect a different inclusion of food byproducts containing TB and CF in the FFPs, such as cocoa hulls and husks. The negative control remained below detection limits for both compounds. To the authors’ knowledge, this is the first study that quantifies TB and CF in FFPs. A previous study quantified plasma TB and CF in postweaning piglets after consumption of 30% salty or sugary FFPs in their diets. The results showed that the TB and CF concentrations were increased (*p* < 0.05) in both groups compared with the control group, which had TB levels below identification limits, suggesting the presence of cocoa-derived products in both salty and sugary FFPs.^27^

Based on the selected inclusion rate of 30% for the target animal categories (ruminants and pigs), TB concentrations in feed containing FFP8 would exceed the EU MLs of 300 mg/kg for ruminants and 200 mg/kg for pigs, whereas feeds formulated with any other FFP remain below these thresholds (Figure 4), provided no additional theobromine sources are incorporated in the diet. An inclusion rate of 26.2% of FFP8 in ruminant feed and 17.4% in pig feed (Table 2) would already bring TB concentrations up to the EU MLs and were then selected for the maximum exposure scenario in ADE calculations.

**Figure 4:**
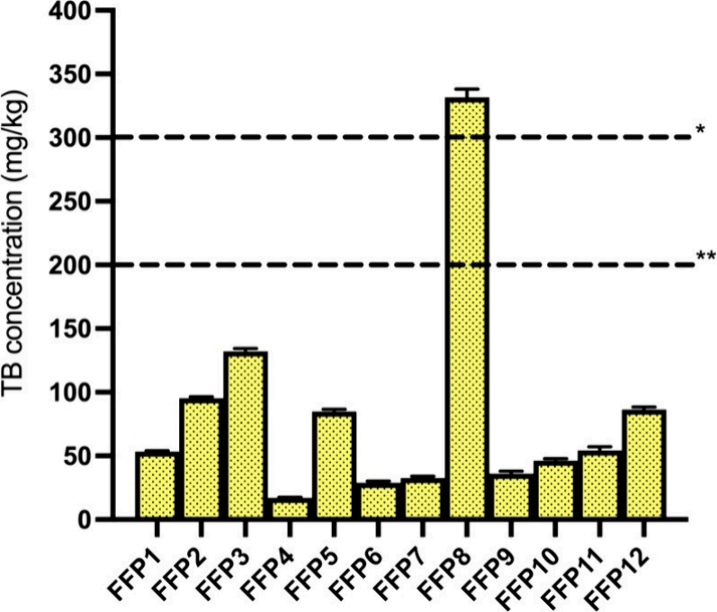
TB levels (mg/kg) in feed with a moisture content of 12%, assuming a 30% on DM basis inclusion rate of the FFPs. *: general TB EU ML (Directive 2002/32/EC) in complete feed, applicable for ruminants; **: TB EU ML (Directive 2002/32/EC) in complete feed for pigs.

### Animal Dietary Exposure Assessment and Safety Considerations

ADE was calculated for each of the four scenarios (minimum, maximum, median, and mean exposure) using the TB and CF concentrations measured in the FFPs and the calculated resulting concentrations per kilogram of DM feed (Table 2). The estimated ADE values for ruminants (dairy cows, cattle for fattening, veal calves, and dairy sheep/goat) and pigs (weaned piglets, pigs for fattening, and lactating sows) are reported in Table 3 for TB and in Table 4 for CF, expressed as mg/kg body weight (bw)/day.

**Table 3:** Estimated TB ADE for Each Animal Species under the Minimum (FFP4), Maximum (FFP8), Median, and Mean Values (across FFP1-FFP12)^b^

			minimum			maximum			median			mean		
species	body weight^a^ (kg)	feed intake^a^ DM (kg/day)	TB (mg/kg of DM feed)	exposure (mg/day)	exposure (mg/kg bw/day)	TB (mg/kg of feed)	exposure (mg/day)	exposure (mg/kg bw/day)	TB (mg/kg of feed)	exposure (mg/day)	exposure (mg/kg bw/day)	TB (mg/kg of feed)	exposure (mg/day)	exposure (mg/kg bw/day)
Ruminants														
dairy cows	650	20	19.45	389.08	0.60	340.91	6818.18	10.49	61.21	1224.13	1.88	91.61	1832.14	2.82
cattle for fattening	400	8	19.45	155.63	0.39	340.91	2727.27	6.82	61.21	489.65	1.22	91.61	732.85	1.83
veal calves	100	1.89	19.45	36.77	0.37	340.91	644.32	6.44	61.21	115.68	1.16	91.61	173.14	1.73
dairy sheep/goat	60	1.2	19.45	23.34	0.39	340.91	409.09	6.82	61.21	73.45	1.22	91.61	109.93	1.83
Pigs														
piglets (weaned)	20	0.88	19.45	17.12	0.86	227.27	200.00	10.00	61.21	53.86	2.69	82.14	72.28	3.61
pigs for fattening	60	2.2	19.45	42.80	0.71	227.27	500.00	8.33	61.21	134.65	2.24	82.14	180.70	3.01
sows, lactating	175	5.28	19.45	102.72	0.59	227.27	1200.00	6.86	61.21	323.17	1.85	82.14	433.68	2.48

a EFSA, 2017;^46^ EFSA, 2024.^47^

b For each species, body weight (kg) and daily DM feed intake (kg/day) are listed. TB concentration (mg/kg of DM feed), daily exposure (mg/day), and normalized exposure to body weight (mg/kg bw/day) are presented under each scenario.

**Table 4:** Estimated CF ADE for Each Animal Species under the Minimum (FFP6), Maximum (FFP8), Median, and Mean Values (across FFP1-FFP12).^b^

			minimum			maximum			median			mean		
species	body weight^a^ (kg)	feed intake^a^ DM (kg/day)	CF (mg/kg of DM feed)	exposure (mg/day)	exposure (mg/kg bw/day)	CF (mg/kg of feed)	exposure (mg/day)	exposure (mg/kg bw/day)	CF (mg/kg of feed)	exposure (mg/day)	exposure (mg/kg bw/day)	CF (mg/kg of feed)	exposure (mg/day)	exposure (mg/kg bw/day)
Ruminants														
dairy cows	650	20	2.97	59.37	0.09	35.09	701.89	1.08	6.59	131.72	0.20	9.94	198.84	0.31
cattle for fattening	400	8	2.97	23.75	0.06	35.09	280.76	0.70	6.59	52.69	0.13	9.94	79.54	0.20
veal calves	100	1.89	2.97	5.61	0.06	35.09	66.33	0.66	6.59	12.45	0.12	9.94	18.79	0.19
dairy sheep/goat	60	1.2	2.97	3.56	0.06	35.09	42.11	0.70	6.59	7.90	0.13	9.94	11.93	0.20
Pigs														
piglets (weaned)	20	0.88	2.97	2.61	0.13	23.40	20.59	1.03	6.59	5.80	0.29	8.97	7.89	0.39
pigs for fattening	60	2.2	2.97	6.53	0.11	23.40	51.47	0.86	6.59	14.49	0.24	8.97	19.73	0.33
sows, lactating	175	5.28	2.97	15.67	0.09	23.40	123.53	0.71	6.59	34.77	0.20	8.97	47.35	0.27

a EFSA, 2017;^46^ EFSA, 2024.^47^

b For each species, body weight (kg) and daily DM feed intake (kg/day) are listed. CF concentration (mg/kg of DM feed), daily exposure (mg/day), and normalized exposure to body weight (mg/kg of body weight/day) are presented under each scenario.

At an inclusion rate of 30% (DM basis) of FFPs in the animals’ diets and considering the minimum, median, and mean occurrence values, pigs exhibit the highest exposure to TB and CF. Specifically, weaned piglets exhibited the highest exposure levels, whereas lactating sows showed the lowest. TB exposure values ranged from 0.59 to 0.86 mg/kg bw/day (minimum), 1.85–2.69 mg/kg bw/day (median), and 2.48–3.61 mg/kg bw/day (mean). As for TB, the same exposure patterns were observed for CF, with piglets showing the highest exposure to CF, approximately 1.5 times that of lactating sows, which showed the lowest.

Ruminants would generally be exposed to lower TB and CF concentrations. TB ranged from 0.30 to 0.60 mg/kg bw/day (minimum), 1.16–1.88 mg/kg bw/day (median), and 1.73–2.82 mg/kg bw/day (mean). As for pigs, also for ruminants, TB and CF show a comparable pattern, with veal calves, dairy sheep/goat, and cattle for fattening showing similar exposure levels, while dairy cows exhibited approximately 1.5 times higher exposure.

In the maximum exposure scenario, the inclusion rate of FFP8 was reduced from the standard 30 to 27.1% for ruminants and to 18.1% for pigs to remain within the EU MLs for TB. In this case, animal exposure corresponds to the levels associated with feed containing TB at the EU ML of 300 ppm (12% DM) for ruminants and 200 ppm (12% DM) for pigs. In this scenario, ruminant TB exposure ranges from 6.44 mg/kg of body weight (bw)/day in veal calves to 10.49 mg/kg of bw/day in dairy cows. Similarly, pig TB exposure ranges from 6.86 to 10.00 mg/kg bw/day. By adjustment of the IR% so that TB concentrations align with the EU MLs, the calculated ADE for ruminants and pigs converged to a more comparable range. Nevertheless, within each species category, dairy cows and weaned piglets still exhibited exposures around 1.5 times higher than those of veal calves and lactating sows, respectively, reflecting differences in the production stage and physiological demands.

This can be explained by relative feed intake, although all animals in the same category receive feed containing the same TB concentration, those with higher daily intake per kilogram of body weight naturally consume higher levels of TB and CF. By dividing feed intake values by body weight values (Table 3), it is clear that dairy cows have high physiological demands^57^ and ingest around 3.1% of their body weight in DM feed, also based on the need to support milk yield, whereas veal calves only consume around 1.9% of their body weight. This higher intake ratio in dairy cows directly translates into higher ADE values. In the case of weaned piglets, they are in a growth stage, demanding even higher intakes, around 4.4% of their body weight, compared to lactating sows that consume comparatively lower amounts (3.1%). Overall, these estimates are based on the assumption that FFPs are the only source of TB and CF in the diet and are based on default body weight and intake parameters; access to a comprehensive EU feed consumption database with real-world values of feed intake or model diets across developmental and physiological stages would enable even more accurate exposure assessments.^58,59^

When the derived ADE values for TB are compared with reported NOAEL or LOAEL values across the selected animal categories, ruminant exposure remains below these levels. In dairy cows, effects on milk production, namely, reduced milk yield and increased fat content, were observed at around 15 mg/kg bw/day of TB for an exposure period of 1 week.^19,60^ No health effects were observed at approximately 23 mg/kg bw/day for up to seven months.^61^ These values remain above the predicted median and mean exposure scenarios (1.88–2.82 mg/kg of body weight (bw)/day), while the maximum exposure estimate (10.49 mg/kg of body weight (bw)/day) is closer to the value at which only changes in milk yield and fat are observed.

For young cattle, adverse effects levels were drawn from a suspected chocolate poisoning report of calves,^62^ in which TB concentrations were estimated and clinical signs (i.e., hyperexcitability, sweating, and increased respiration and heart rate) were reported at 45–90 mg/kg bw/day over a period of several weeks. All modeled exposures for veal calves (0.37–6.44 mg/kg bw/day) remained well below these levels, although a definitive no-effect dose has not been established. A more recent report of suspected poisoning in dairy cattle supplemented with various amounts of chocolate chips was published in 2021,^11^ but chocolate chips and theobromine intake at the time of the incident were unknown.

In goats, reduced voluntary intake and body weight gain occurred at the lowest theobromine tested level following the inclusion of cocoa shell or cocoa dust into goat feed for 56 days,^63^ assumed to be approximately 300 mg/kg bw/day.^19^ More recent findings confirmed reduced feed intake at TB intakes of 1.51 g/head per day from the inclusion of cocoa bean shell in the diet for 31 days,^64^ corresponding approximately to 25.2 mg/kg bw/day, for a 60 kg lactating goat, without affecting milk yield, as well as milk fat, protein, and casein contents. In other studies in lactating ewes, approximate exposure to TB at 9–10 mg/kg bw/day for 21 days^65^ and 7.25 mg/kg bw/day for 35 days,^66^ derived from cocoa bean shells and husk, respectively, did not have adverse effects on DM intake, milk yield, and fat and protein percentage. Overall, in the case of modeled ADE in sheep and goats (0.39–6.82 mg/kg bw/day), the ADE remained below these no-observed adverse effect levels.

In pigs, ingestion of cocoa meal corresponding to 24 mg/kg bw/day of TB for 126 days resulted in lethargy and growth retardation, most evident during the initial 4 to 5 weeks.^67^ A NOAEL of 7 mg/kg bw/day for young growing pigs was identified by the EFSA Panel^19^ based on reduced piglet growth performance,^14^ when dried whey was substituted with a dried byproduct of milk chocolate, candy, and food industries, over a period of 35 days. TB concentrations in the substitute were not known and assumed to be 1.35 g/kg based on literature data. A study published in 2017 by Ogunsipe and colleagues studied the growth performance of weaning pigs on a dietary cocoa bean shell with declared TB content in an 84-day feeding trial. Animal feed intake was not affected, but live weight gain decreased when TB exposure exceeded approximately 29 mg/kg bw/day, while feed conversion was poorer at approximately 14 mg/kg bw/day, which was the lowest tested dose.^68^

Older growing pigs appeared to tolerate somewhat higher TB exposures.^19^ This is also supported by a more recent study^69^ in which dietary inclusion of cocoa husks for 21 days in large white pigs did not affect feed intake, weight gain, or feed efficiency. Although TB concentrations in the cocoa husks were not reported, assuming concentration levels reported by Carta and colleagues,^66^ the estimated TB exposure without adverse effects would be approximately 9 mg/kg bw/day.

In summary, modeled ADE scenarios including the minimum, median, and mean exposure (0.86–3.61 mg/kg bw/day) remained below the reported toxicological threshold values, whereas the maximum exposure scenario for piglets (10.49 mg/kg bw/day) exceeded the established NOAEL value of 7 mg/kg bw/day, suggesting higher susceptibility of this animal category when fed at the EU MLs.

These results underline the need for species-specific evaluation of diet formulation and TB quantification in FFPs before their inclusion in animal diets.

Furthermore, given the increasing interest in incorporating food co- and byproducts into animal feed, additional studies in which conventional feed is replaced by cocoa-derived or cocoa-containing ingredients, even when assessing different endpoints or aspects of animal production, would be extremely valuable if TB concentrations in both the original matrix and the feed, as well as actual feed intake and animal body weights, are consistently included. Such comprehensive reporting would facilitate more accurate safety assessments and support the refinement of toxicological thresholds.

Overall, the findings contribute to a growing body of evidence supporting the safe and sustainable use of FFPs in animal nutrition, reinforcing their role as a viable strategy for reducing food waste and enhancing the environmental efficiency of livestock production systems.

## Abbreviations Used

ADE: animal dietary exposure
aNDFom: amylase neutral detergent fiber on an organic matter basis
BSE: bovine spongiform encephalopathy
bw: body weight
CF: caffeine
CP: crude protein
DM: dry matter
EC: European Commission
EE: ether extract
EFSA: European Food Safety Authority
EU: Europe
FFP: former food product
FI: feed intake
FLW: food losses and food waste
HACCP: Hazard Analysis and Critical Control Point
HPLC-UV: high-performance liquid chromatography coupled with ultraviolet detection
HPTLC: high-performance thin-layer chromatography
IR: inclusion rate
LOAEL: lowest-observed adverse effect
LOD: the limit of detection
LOQ: the limit of quantification
MLs: maximum levels
NC: negative control
NDF: neutral detergent fiber
NOAEL: no-observed adverse effect
PC: positive control
Rf: retention factor
Rt: retention time
SD: standard deviation
TB: theobromine
